# MOG-induced experimental autoimmune encephalomyelitis in the rat species triggers anti-neurofascin antibody response that is genetically regulated

**DOI:** 10.1186/s12974-015-0417-2

**Published:** 2015-10-29

**Authors:** Sevasti Flytzani, Andre Ortlieb Guerreiro-Cacais, Marie N’diaye, Maren Lindner, Christopher Linington, Edgar Meinl, Pernilla Stridh, Maja Jagodic, Tomas Olsson

**Affiliations:** Center for Molecular Medicine, Department of Clinical Neuroscience, Karolinska Institutet, CMM L8:04 Stockholm, Sweden; Institute of Infection, Immunity and Inflammation, University of Glasgow, Glasgow, UK; Institute of Clinical Neuroimmunology, Ludwig-Maximilians-Universität, Munich, Germany

**Keywords:** Neurofascin, EAE, MOG, MBP_63–88_, Epitope spreading, MS, Genetic regulation

## Abstract

**Background:**

Ιn multiple sclerosis (MS), axonal damage leads to permanent neurological disabilities and the spreading of the autoimmune response to axonal antigens is implicated in disease progression. Experimental autoimmune encephalomyelitis (EAE) provides an animal model that mimics MS. Using different EAE models, we investigated the pathophysiological basis of epitope spreading to neurofascin, a protein localized at the node of Ranvier and its regulation by non-MHC genes.

**Methods:**

We used two different EAE models in DA rat; one which is induced with myelin oligodendrocyte glycoprotein (MOG) which leads to disease characterized by profound demyelination, and the second which is induced with myelin basic protein (MBP) peptide 63–88 which results in severe central nervous system (CNS) inflammation but little or no demyelination. We determined anti-neurofascin antibody levels during the course of disease. Furthermore, the anti-neurofascin IgG response was correlated with clinical parameters in 333 (DAxPVG.1AV1) x DA rats on which we performed linkage analysis to determine if epitope spreading to neurofascin was affected by non-MHC genes.

**Results:**

Spreading of the antibody response to neurofascin occurred in demyelinating MOG-induced EAE but not in EAE induced with MBP peptide 63–88. Anti-neurofascin IgG levels correlated with disease severity in (DAxPVG.1AV1) x DA rats, and a genomic region on chromosome 3 was found to influence this response.

**Conclusions:**

Inter-molecular epitope spreading to neurofascin correlates with disease severity in MOG-EAE is dependent on extensive demyelination and is influenced by non-MHC genes. The findings presented here may shed light on factors involved in the severity of MS and its genetics.

**Electronic supplementary material:**

The online version of this article (doi:10.1186/s12974-015-0417-2) contains supplementary material, which is available to authorized users.

## Background

Multiple sclerosis (MS) is a chronic inflammatory disease characterized by demyelination and axonal damage leading to chronic neurological deficits. A role of B cells and autoantibodies in disease pathogenesis is supported by numerous observations derived from a wide range of clinical, pathological, and experimental studies. In particular, oligoclonal bands (OCB) are found in the cerebrospinal fluid (CSF) in the majority of MS patients, implying involvement of intrathecal synthesis of immunoglobulin and B cells [1] even though the antigenic specificity of this response remains unclear [2]; plasma exchange can lead to disease amelioration in some patients [3]; histopathological studies demonstrate that many lesions are associated with immunoglobulin and complement deposition [4]; ectopic B cell follicles are present in the meninges of patients with secondary progressive MS and are implicated in driving disease activity in the underlying parenchyma [5, 6]; demyelinating and axopathic autoantibodies can be identified in patient sera; and finally, phase II studies with anti-CD20 antibodies targeting B cells, such as Rituximab, have a profound effect on disease activity in patients with relapsing-remitting disease [7].

Axonal injury and loss are now recognized to be the cause of permanent neurological disability in MS [8, 9], but the molecular mechanisms responsible for this axonal pathology remain unclear. Evidence is now available implicating contributions from invading inflammatory cells [10], complement [11], glutamate excitotoxicity [12], loss of trophic support provided by myelinating oligodendrocytes [13], and axopathic autoantibodies [14]. The relative contribution of these pathomechanisms in MS remains controversial but using a proteomics-based approach, Mathey et al. identified neurofascin as a potential target for an axopathic autoantibody response [14]. Anti-neurofascin antibody titers were more pronounced in secondary progressive MS than in relapsing-remitting MS, and adoptive transfer of neurofascin-specific antibodies caused rapid worsening of experimental autoimmune encephalomyelitis (EAE), an animal model for MS. This clinical effect was associated with deposition of neurofascin-specific antibody and complement at nodes of Ranvier resulting in inhibition of neurotransmission and axonal damage [14].

Neurofascin is just one among the increasing number of myelin and non-myelin antigens now implicated in the pathogenesis of MS, but the mechanisms by which these autoimmune responses are initiated are still elusive. Attempts at resolving this problem are confounded by epitope spreading, the ability of the initial pathogenic response to one autoantigen to initiate secondary responses to other tissue-specific targets. This phenomenon has been described extensively in EAE, in which epitope spreading is associated with induction of clinical relapses [15]. In the case of neurofascin, we hypothesized epitope spreading to involve this autoantigen would be most likely in disease models in which there is extensive demyelination and axonal injury. This is not the case in the classical models of EAE induced by immunization with myelin basic protein (MBP) or MBP peptides, a protocol that results in a monophasic neuroinflammatory disease in rats that is associated with little or no demyelination [16, 17]. In contrast, immunization with myelin oligodendrocyte glycoprotein (MOG) leads to a relapsing disease with extensive demyelination [18, 19], a situation predicted to enhance availability of neurofascin epitopes to elicit a secondary response. We therefore investigated if there was a difference in epitope spreading to neurofascin in these two models of EAE in DA rat and if there was any correlation with disease severity. We report that the induction of EAE using recombinant MOG is associated with the development of a neurofascin-specific autoantibody response, which is not observed in animals with MBP peptide 63–88 induced EAE. Screening for this response in a (DAxPVG.1AV1) x DA (hereafter referred as DA backcross) population immunized with MOG revealed that neurofascin-specific antibody levels which correlate with disease severity and that this antibody response was dependent on non-MHC genes. These observations are of great importance in view of the current attempts to extend the study of MS genetics to explore mechanisms determining disease severity.

## Methods

### Ethics statement

All experiments in this study were approved and performed in accordance with the guidelines from the Swedish National Board for Laboratory Animals and the European Community Council Directive (86/609/EEC) under the ethical permits N338/09, N298/11, and N478/12 entitled “Genetic regulation, pathogenesis and therapy of EAE, an animal model for multiple sclerosis”, which was approved by the North Stockholm Animal Ethics Committee (Stockholms Norra djurförsöksetiska nämnd). The rats were tested according to a health-monitoring program at the National Veterinary Institute (Statens Veterinärmedicinska Anstalt, SVA) in Uppsala, Sweden.

### Animals

The DA rats were purchased from Harlan Laboratories (Harlan Laboratories, Venray, The Netherlands). The animals were housed in polystyrene cages containing aspen wood shavings and had free access to standard rodent chow and water. They were immunized with either MOG or MBP_63–88_ for induction of EAE and used for T cell proliferation assays ( see the “Thymidine incorporation assay” for description). Additionally, a DA backcross population was established between DA/Kini and MHC-identical PVG.1AV1 strains, as described previously [20]. To create the F1 generation, four breeding pairs with DA female founders were established. The N2 generation was bred from eight breeding pairs, with DA females or males crossed to F1 males and females, respectively. Four N2 litters, 421 backcross rats (213 females and 208 males) were subjected to MOG-EAE induction. Assessment of antibodies against neurofascin at day 35 after MOG immunization was performed in 333 (174 females and 159 males) DA backcross rats as 88 of the DA backcross rats either died during EAE or sacrificed according to the ethical considerations.

### Induction and determination of disease phenotypes

DA female age-matched rats between 11 and 12 weeks of age were anesthetized with isoflurane (Forene; Abbott Laboratories, Chicago, IL, USA) and immunized by a single subcutaneous injection in the dorsal base of the tail with 200 μl inoculum containing either one of the following five preparations: (a) 20 μg rat recombinant MOG (aa 1–125 from the N terminus, expressed in *Escherichia coli* and purified to homogeneity by chelate chromatography, hereafter denoted MOG) in phosphate-buffered saline (PBS) (Life Technologies, Paisley, Scotland) emulsified (1:1) with incomplete Freund’s adjuvant (IFA) (Sigma-Aldrich, St Louis, MO, USA), (b) 100 μg guinea pig MBP peptide (aa 63–88 from the N terminus with sequence AARTTHYGSLPQKSQRSQDENPWHF, hereafter denoted MBP_63–88_) purchased from GL Biochem (Shanghai) Ltd. (Shanghai, China) in PBS emulsified (1:1) with complete Freund’s adjuvant (CFA) (Sigma-Aldrich, St Louis, MO, USA) containing 0.5 mg *Mycobacterium tuberculosis* (MTB) (DIFCO Laboratories, Detroit, Michigan, USA), (c) 50 μg recombinant rat neurofascin 155 (hereafter denoted rrNF) (R&D systems, Minneapolis, MN, USA) in PBS emulsified (1:1) with CFA containing 1 mg MTB, (d) IFA, or (e) CFA containing 0.5 mg *M. tuberculosis*.

In another experiment, female and male DA backcross rats between 8 and 16 weeks of age were anesthetized with isoflurane and each animal received a 200-μl inoculum containing MOG (females 12.5–40 μg and males 25–80 μg) in PBS emulsified (1:1) with IFA.

The rats were weighed and monitored daily for clinical signs of EAE, from day 7 until the day of sacrifice at day 56 post immunization (p.i.) for the DA rats and day 35 p.i. for the DA backcross rats. The clinical scoring scale was as follows: 0, no clinical signs of EAE; 1, tail weakness or tail paralysis; 2, hind leg paraparesis or hemiparesis; 3, hind leg paralysis or hemiparalysis; 4, tetraplegia or moribund; and 5, death. The following clinical parameters were used: EAE incidence, clinical signs for more than 1 day; onset of EAE, the first day clinical signs were observed; maximum EAE score, the highest clinical score observed during disease; cumulative EAE score, the sum of all daily clinical scores; duration of EAE, the number of days with EAE; and weight loss (WL0), which is a quantitative trait considered to correlate well with a clinical EAE course and that represents (weight at day 0 p.i.—minimum weight during the experiment)/weight at day 0 p.i.

### Serum collection

To determine antibody levels, blood was collected for serum at day 12, day 26, day 41, and day 56 p.i. in the DA rats and at day 12 and 35 p.i. in the DA backcross rats. The rats were anesthetized under isoflurane, and the blood was collected by tail bleeding; at the day of sacrifice, the rats were euthanized under CO_2_ and the blood was collected through cardiac puncture. The blood was centrifuged at 3600 rpm at 4 °C for 20 min, and serum was collected and preserved at −20 °C until ELISA analysis.

### Anti-neurofascin IgG and IgG isotypes determination

ELISA was used to determine anti-rrNF IgG and isotype-specific for IgG1 and IgG2b, anti-MOG IgG, and anti-MBP_63–88_ IgG. Furthermore, to assess specificity of anti-rrNF IgG antibodies, we measured anti-human neurofascin 155 (hNF155), anti-human neurofascin 186 (hNF186), anti-C-reactive protein (CRP) IgG and anti-interleukin 9 receptor (IL-9R) IgG by ELISA. To investigate cross-reactivity between anti-rrNF IgG and anti-contactin-2 (TAG-1), anti-TAG1 IgG antibodies were assessed by the same method.

Ninety-six-well ELISA plates (Sigma-Aldrich, Roskilde, Denmark) were coated with 10 μg/ml of either of the following: rrNF (R&D systems), hNF155, hNF186 (both produced as described in Ng et al. Neurology 2012), recombinant rat MOG, guinea pig MBP_63–88_, and recombinant rat TAG-1 (R&D systems), each one of them diluted in 0.1 M NaHCO3 (pH 8.2) (100 μl/well). The coated plates were stored overnight at 4 °C. The plates were washed twice with PBS/0.05 % Tween-20, and free binding sites were blocked with 5 % fat-free milk in PBS/0.05 % Tween-20 for 1 h at room temperature (RT). The rat sera was diluted 1:200 (for anti-rrNF IgG, IgG1, and IgG2b isotypes, for anti-MOG IgG in MBP_63–88_-EAE rats, for anti-MBP_63–88_ IgG in MOG-EAE rats, for anti-hNF155 IgG, for anti-hNF186 IgG, for anti-CRP IgG, and for anti-IL-9R IgG), 1:1000 (for anti-MOG IgG in MOG-EAE rats and for anti-MBP_63–88_ IgG in MBP_63–88_-EAE rats) in PBS/1 % milk and incubated in duplicates in plates for 1 h at RT. The plates were washed and incubated for 1 h at RT with rabbit anti-rat IgG (1:2000), IgG1 (1:1000), and IgG2b (1:2000) (Nordic, Tilburg, The Netherlands). Unbound antibodies were removed by washing prior to addition of a peroxidase-conjugated goat anti-rabbit antiserum (Nordic) diluted in PBS/0.05 % Tween-20 (1:10,000). After 30 min of incubation, the plates were washed extensively and bound antibodies were visualized with 3,3′,5,5′-tetramethylbenzidine (Sigma). The reaction was stopped by addition of 1 M HCl after 5–10 min in darkness, and the optical density (OD) was read at 450 nm using an Emax Microplate Reader (Molecular Devices, Sunnyvale, USA). Seven twofold dilutions of serum samples from rats with high levels of each antibody detected in a pilot study were included on each plate to create a standard curve and to permit interplate comparisons. The serum samples from the animals immunized with IFA, CFA, or rrNF were also included in each ELISA plate as negative (serum from IFA- and CFA-immunized rats) and positive controls (serum from rrNF-immunized rats).

In the MOG- and MBP_63–88_-immunized DA rats, we considered the serum specimens to be antibody-positive when the optical density exceeded the cut-off value, which was set at 5 standard deviations (SD) above the mean optical density in serum specimens from IFA- or CFA-immunized DA rats, respectively.

In the DA backcross population, due to the absence of IFA-immunized control animals, we arbitrarily chose to use a more stringent cut-off for antibody positivity. We considered serum specimens to be antibody-positive when OD exceeded the cut-off value, which was set at 15 % of the serum specimens exhibited the highest antibody values. The stringent threshold was also based on testing different cut-off values, e.g., when the serum specimens were considered positive if they exceeded 10 or 20 % of specimens with the highest OD values, and the outcome in our analysis did not differ. Furthermore, OD values lower than the cut-off of 15 % that we set in the analysis clearly represented a background signal.

### Adsorption of anti-rrNF IgG antibodies

To examine if there is cross-reactivity between anti-rrNF IgG and anti-TAG1 IgG antibodies, we depleted anti-rrNF antibodies in selected serum samples by performing consecutive adsorptions to plate-bound rrNF. Subsequently, we compared the response against TAG-1 between intact serum and anti-rrNF IgG antibody-depleted serum.

### Tissue collection and preparation

To determine T cell response, 28 days after MOG immunization, the DA rats were euthanized under CO_2_ and the spleen and deep cervical lymph nodes were collected. The spleen and lymph nodes were placed in DMEM (Gibco-BRL, Grand Island, NY), enriched with 10 % fetal calf serum, 1 % glutamine, 1 % penicillin-streptomycin, and 1 % pyruvic acid (all from Life Technologies, Paisley, Scotland) before being mechanically separated by passing through a mesh screen with the bolus of a syringe. Erythrocytes were removed from the spleen samples by lysis with ACK buffer (Gibco, Invitrogen, Karlsruhe, Germany). The samples were resuspended in PBS, and cell numbers were assessed by counting with trypan blue as a dead cell exclusion dye.

### Thymidine incorporation assay

To assess T cell proliferation, 400,000 splenocytes/well and 100,000 deep cervical lymph node cells/well were plated in 96-well round bottom plates (Nunc, Roskilde, Denmark) in triplicates. The cells were stimulated with either of the following: medium, 20 μg/ml MOG, 20 μg/ml rrNF (R&D systems), or 1 μg/ml concanavalin A (ConA) (Sigma-Aldrich) for 72 h at 37 °C and 5 % CO_2._ Eighteen hours before collection, 1 mCi of H^3^ radioactive thymidine (GE Halthcare, Bucks, UK) was added to each well. The cells were harvested using a Wallac Tomtec (Perkin, Elmer, Waltham, USA) and isotope incorporation was measured using a Wallac TriLux 1450 Microbeta (Perkin Elmer).

### Genotype

Genotyping of 333 DA backcross rats was performed on DNA extracted from the tail tip/ear according to a standard protocol. Genotypes were determined by PCR amplification of 137 microsatellite markers. Fluorophore-conjugated primers were used (Applied Biosystems, Eurofins MWG, Operon, Foster City, CA, USA), and PCR products were size fractionated using an electrophoresis capillary sequencer (ABI3730, Applied Biosystems). The genotypes were analyzed using the GeneMapper software (v. 3.7, Applied Biosystems), and two independent observers manually evaluated all genotypes.

### Statistical analysis

Spearman’s rank correlations between antibody levels and clinical EAE phenotypes or between antibody levels against other antigens were calculated in GraphPad Prism 5.0 (Graph Pad, San Diego, CA, USA). Differences in T cell response between medium and neurofascin stimulation were calculated in GraphPad Prism 5.0 using non-parametric Mann-Whitney *U* test. In the DA backcross population, antibody levels between healthy and sick groups and EAE clinical parameters between anti-neurofascin IgG(−) and anti-neurofascin IgG(+) groups were compared using non-parametric Mann-Whitney *U* test in GraphPad Prism 5.0.

### Linkage analysis

The genetic map was defined using publicly available genome sequence (http://oct2012.archive.ensembl.org/index.html). Linkage analysis was performed with the statistical software R/qtl version 2.11.1. [21]. A single-quantitative trait loci (QTL) model analysis was performed using Haley-Knott regression model on transformed OD values, and “weight at day 0” and “sex” were used as additive covariates. Permutation tests (*N* = 1000) were performed to determine the threshold levels for significant linkage. Differences in allelic effects of the QTL were analyzed with the non-parametric Mann-Whitney *U* test using GraphPad Prism 5.0.

## Results

### Epitope spreading to neurofascin occurs in MOG-EAE

To assess whether an antibody response to neurofascin could arise secondary to a response to another central nervous system (CNS) antigen, we used the two most prevalent EAE models in DA rats, namely immunization with MOG in IFA and with MBP_63–88_ in CFA.

**Fig. 1:**
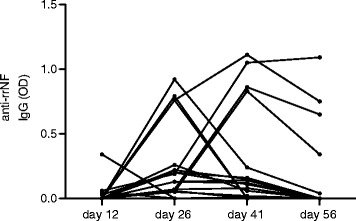
Anti-rrNF IgG response over time in DA rats in MOG-EAE. Thirty-one DA rats were immunized with MOG in IFA, and anti-rrNF IgG levels were assessed in the sera at different time points (day 12, day 26, day 41, and day 56) after immunization by ELISA. The sera were diluted 1:200. Data are presented in OD values. Only anti-rrNF IgG(+) rats are included in the graph

All rats immunized with MOG developed a MOG-specific IgG response. This was already detected at day 12 p.i. and persisted throughout the course of the disease (Additional file 1: Table S1). Anti-rrNF IgG antibodies could also be detected in these sera, but only at a late stage of MOG-induced EAE; 16 out of 31 (52 %) MOG-immunized rats were seropositive for rrNF-specific IgG antibodies at day 26 p.i. In most of these rats, this anti-rrNF antibody response persisted until day 41 p.i. and in some cases, titers remained high until the rats were sacrificed at day 56 p.i. In four animals, rrNF-specific antibodies were already detected on day 12, but the titers were very low in three of them, implying that epitope spreading tends to occur during later phases of disease (Fig. 1 and Additional file 1: Table S1). In contrast, fewer rats, 10 out of 31 (32 %), with MOG-induced EAE developed an antibody response to MBP_63–88_. Moreover, this low titer response was generally only observed at single time points, with the exception of two rats with high levels at two time points (Additional file 1: Table S1). Interestingly, anti-rrNF IgG response did not correlate with anti-MBP_63–88_ IgG response (Additional file 2: Figure S1).

**Fig. 2:**
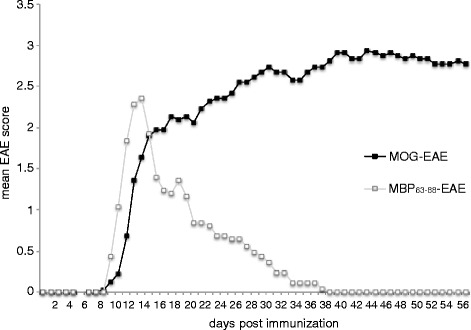
EAE course in MOG- and MBP_63–88_-immunized DA rats. The DA rats were immunized with 20 μg recombinant MOG in PBS emulsified (1:1) in IFA or 100 μg guinea pig MBP_63–88_ peptide in PBS emulsified (1:1) with CFA containing 0.5 mg MTB. The rats were monitored daily for clinical signs of EAE, from day 7 until the day of sacrifice, at day 56 after immunization

In contrast to MOG-induced EAE, we observed little or no antibody to rrNF in the DA rats with MBP_63–88_-induced EAE (Additional file 3: Table S2). All MBP_63–88_-immunized rats developed an acute monophasic disease with symptoms reaching maximum severity around day 12 p.i., after which the animals slowly recovered over the next 10 to 14 days (Fig. 2). All animals developed high titer antibody responses to MBP_63–88_ but only one developed a transient, low titer response to rrNF (Additional file 3: Table S2). Epitope spreading to MOG was somewhat more pronounced in that 16 % (4 of 25) of the rats immunized with MBP_63–88_ developed a MOG-specific IgG response by day 56 p.i. (Additional file 3: Table S2).

### Validation of anti-neurofascin reactivity

To validate the data we obtained using a commercially available rrNF, we screened the rat sera for reactivity to hNF155 and hNF186. This revealed a similar pattern of reactivity to all three neurofascin variants (Additional file 4: Figure S2a and b). Furthermore, to eliminate the possibility that this response might include a significant component directed against their C-terminal histidine tags, we compared reactivity to rrNF and two other recombinant proteins generated using the same cell line and carrying an identical histidine tag (CRP and IL-9R; R&D Systems). Low levels of reactivity to CRP and IL-9R were detected in some animals but their correlation to the response to rrNF was minimal (Additional file 4: Figure S2c and d) implying the response to rrNF does not include a significant component directed against its histidine tag.

Nonetheless this does not exclude the possibility that the antibody response to rrNF cross reacts with other members of the Ig superfamily of cell adhesion molecules; one candidate being TAG-1, a potential MS autoantigen [22] that exhibits approximately 30 % sequence similarity to neurofascin. To check for cross-reactivity with TAG-1, we first depleted MOG-EAE rat serum of anti-rrNF IgG antibodies by consecutive adsorptions to plate-bound rrNF and then, investigated the reactivity against TAG-1 using non-depleted serum as control. We observed no reactivity against TAG-1 in the anti-rrNF IgG-depleted samples whereas non-depleted samples displayed anti-TAG-1 IgG response (Additional file 5: Figure S3), implying that antibodies against neurofascin can also bind to TAG-1.

Taken together, our experiments show that detection of anti-rrNF IgG antibodies as measured by ELISA is specific in that the response cross-reacts with hNF155 and hNF186 and does not bind the recombinant proteins His-Tag. However, our data suggests that this antibody response cross-reacts with another nodal antigen, TAG-1.

### MOG-induced EAE is associated with T cell reactivity to rrNF

**Fig. 3:**
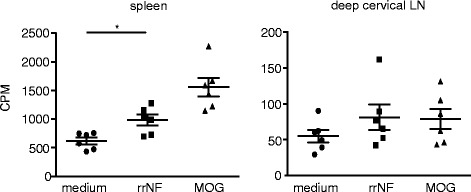
T cell response in DA rats 28 days after MOG immunization. The DA rats were immunized with 20 μg MOG and 28 days later, the spleen and deep cervical lymph nodes were excised and the cells stimulated with a medium, rrNF, and MOG. T cell proliferation was measured by H^3^ thymidine incorporation and displayed as scintillation (*CPM* counts per minute). The statistical significance in the T cell response between the medium and rrNF was calculated by using non-parametric Mann-Whitney test in GraphPad Prism 5.0. *p < 0.05. *Error bars* represent standard error of the mean (SEM)

T cells facilitate B cell responses and are important for the induction of immunoglobulin isotype switch. In order to examine if there is a neurofascin-specific T cell response in the MOG-immunized animals, we collected the spleen and deep cervical lymph nodes of the DA rats 28 days after MOG immunization. The cells were stimulated with a medium, MOG, rrNF, or ConA (as a positive control), and T cell proliferation was measured by thymidine incorporation. Splenocytes from the MOG-immunized donors exhibited a significant recall response to rrNF (*p* < 0.05) (Fig. 3), confirming that MOG-induced EAE is associated with low, but significant T cell reactivity to neurofascin.

### Epitope spreading to involve neurofascin is genetically regulated and is associated with more severe clinical disease

**Fig. 4:**
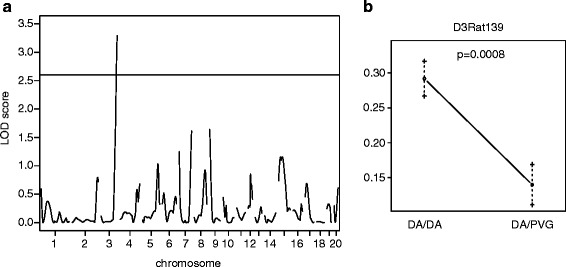
Anti-rrNF IgG2b isotype-specific response is genetically regulated. **a** LOD plot for QTL linkage analysis using Haley-Knott regression on OD values for anti-rrNF IgG2b antibodies at day 35 p.i. in 174 female DA backcross rats. **b** Allelic effect plot at the peak marker of QTL linked in the female DA backcross population. Threshold indicated by the black line for 95 % significant linkage is 2.6, and LOD score is 3.3. *p* value given in the allelic effect plot was calculated using non-parametric Mann-Whitney test in GraphPad Prism 5.0

**Fig. 5:**
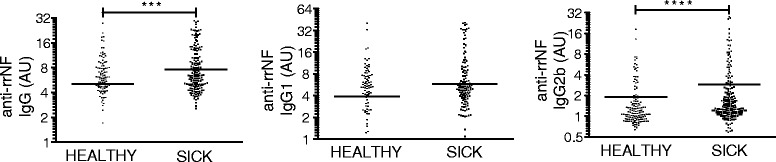
Anti-rrNF IgG and IgG1 isotype-specific and IgG2b isotype-specific reactivity in 333 DA backcross rats. Anti-rrNF IgG and IgG1 isotype-specific and IgG2b isotype-specific levels were assessed in serum at day 35 after MOG immunization in the 333 DA backcross by ELISA, of which 136 rats were healthy and 197 were sick. Sera were diluted in 1:200. Data are presented in arbitrary units (AU). The statistical significance between the healthy and sick rats was calculated by using non-parametric Mann-Whitney test in GraphPad Prism 5.0. ***p < 0.001; ****p < 0.0001. The *line represents* the mean value

**Fig. 6:**
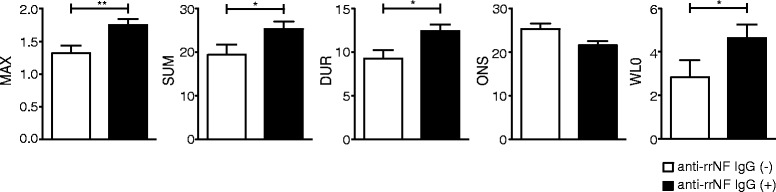
Clinical characteristics of 333 DA backcross rats with present or absent anti-rrNF IgG antibodies. Maximum score (MAX), sum of all scores (SUM), day of onset of the disease (ONS), duration of the disease in days (DUR), and weight change compared to weight at day 0 (WL0) in the 333 DA backcross rats. *Anti-rrNF IgG (−/+)* absence/presence of anti-rrNF IgG. The statistical significance between anti-rrNF IgG(−) and IgG(+) rats was calculated by using non-parametric Mann-Whitney test in GraphPad Prism 5.0. *p < 0.05; **p < 0.01. *Error bars* represent SEM

Our primary data indicate that MOG-induced EAE is associated with epitope spreading leading to the development of a potentially pathogenic autoantibody response. To determine whether this phenomenon was genetically regulated. we immunized 333 rats from a DA backcross population with MOG in IFA of which 197 developed clinical disease. This not only allowed us to perform linkage analysis with respect to the rrNF-specific autoantibody response but also provided sufficient numbers of animals to explore potential correlations between this antibody response and disease severity. Linkage analysis of 174 female DA backcross rats at day 35 identified a QTL on chromosome 3 with a logarithm of the odds (LOD) score of 3.3 that is associated with the rrNF-specific IgG2b response (Fig. 4a). Further analysis plotting the allelic effect at the peak marker (D3Rat139) of the QTL confirmed that DA susceptibility alleles were responsible for driving this response (Fig. 4b). The confidence interval of the chromosome 3 QTL covers 70 to 161 Mb and, therefore, harbors a large number of genes that might be responsible for this effect on epitope spreading to involve neurofascin in MOG-induced EAE. Neurofascin-specific IgG, IgG1 (Th2 associated [23, 24]) and IgG2b (Th1 associated [23]) levels were assessed by ELISA using the sera collected at day 35 p.i. This revealed that anti-rrNF IgG and IgG2b responses were significantly higher in the diseased compared to the healthy DA backcross rats (IgG 7.59 ± 0.39 vs. 5.1 ± 0.42, *p* < 0.001; IgG2b 2.9 ± 0.25 vs. 1.9 ± 0.19, *p* < 0.0001) (Fig. 5). Furthermore, this rrNF-reactive IgG response in animals with EAE was associated with increased disease severity as demonstrated using maximum (MAX) (*p* < 0.01) and cumulative disease (SUM) scores (*p* < 0.05), disease duration (DUR) (*p* < 0.05), and greater weight loss (WL0) (*p* < 0.05) (Fig. 6). However, no effect on time of disease onset was seen, an observation compatible with the involvement of this response at later stages of disease (Fig. 6). Furthermore, the rats with anti-rrNF IgG2b isotype-specific reactivity exhibited a trend (*p* < 0.1) for higher maximum score compared to rats that had no anti-rrNF IgG2b response (Additional file 6: Figure S4). The difference in rrNF-specific IgG1 antibodies was not statistically significant between these two groups (Fig. 6).

## Discussion

Epitope spreading has been suggested to be responsible for relapses in EAE and also leads to increased disease severity and chronicity [15]. While this concept has been generally discussed with respect to T cell reactivities, we hereby demonstrate that this extends to the B cell response in the MOG-induced EAE model, that this response correlates with disease severity, and that is genetically regulated. While we do not address the relapse aspect, our data strongly supports that the presence of an anti-neurofascin response correlates with heightened disease severity, implying that either anti-neurofascin response might contribute to pathology directly or that simply epitope spreading to neurofascin is a reflection of an exacerbated disease per se. While MS is characterized by both demyelination and direct damage to axons/neurons [25], it is currently not completely clear what leads to axonal injury. Epitope spreading to axonal antigens, among other factors, might account for the neurodegenerative processes and axonal pathology seen in the late, progressive forms of the disease. Such a scenario is consistent with the previous observations of a more frequent presence of anti-neurofascin antibodies in progressive MS [14].

Epitope spreading likely results from the loss of self-tolerance [26] driven by tissue damage resulting in increased availability of autoantigens in an inflammatory context. Our data support this concept in that little or no spread to MOG or rrNF-specific B cell responses was observed in the animals with MBP_63–88_ induced disease. The fact that disease in the MBP model is transient prevents us from drawing more extensive conclusions in this respect since chronic inflammation in the absence of demyelination might also result in epitope spreading. Nonetheless, it is attractive to consider that extensive demyelination in an inflammatory context such as seen in MOG-induced EAE is required to break/disrupt self-tolerance to neurofascin and other myelin-associated autoantigens.

Neurofascin exists in two isoforms: neurofascin 186 (NF186), a neuronal protein that clusters with voltage-gated Na + channels at the node of Ranvier and plays an important role in saltatory conduction; neurofascin 155 (NF155), an oligodendroglial/myelin protein that maintains the structure of the paranodal junctional complex [27]. In the present study, we do not distinguish between these two isoforms although our data demonstrate that both are recognized by components of the rrNF-reactive repertoire. Previous studies demonstrate that such antibodies are able to mediate axonal injury by binding to NF186 exposed at the node of Ranvier [6, 14], but whether this mechanism contributes significantly to enhance disease activity in seropositive animals in our study is an open question. Furthermore, we have observed that the antibody response to rrNF cross-reacts with another member of the Ig superfamily, TAG-1, a recently identified target in MS, which elicits an encephalitogenic T cell response selectively targeting gray matter tracts in experimental animals [22]. If the immune response to these proteins does overlap, it might mean that together they constitute a molecular hot spot for the development of autoaggressive responses targeting the nodal domains of myelinated axons, but at the same time raises the question as to which might be the primary or dominant target.

It should also be noted that while our data in the DA backcross population demonstrate a significant association between the presence of rrNF-specific IgG/IgG2b responses and disease severity, this may simply reflect that epitope spreading to neurofascin is a consequence rather than a cause of exacerbated disease.

However, irrespective of the pathological significance of the rrNF-specific response in animals with MOG-EAE, our genome-wide linkage analysis demonstrates that this is at least in part controlled by a QTL on chromosome 3 that regulates the rrNF-specific IgG2b response. This technique does not allow fine-mapping down to the level of single genes and requires validation. However, this locus on chromosome 3 overlaps with a QTL we demonstrated previously to regulate EAE severity and the MOG-specific IgG response during the later phase of EAE in the same DA backcross population [20, 28]. Similarly in a (DAxPVG) x PVG backcross study, this locus was linked to the MOG-specific IgG2c response and disease severity [20, 28], while the same QTL showed a strong linkage to disease severity and MOG-specific IgG and IgG2b levels in the 10th generation of a DAxPVG advanced intercross line [20, 28]. In rats, IgG2b and IgG2c are associated with the activation of Th1 dependent responses [23] that actively participate in the induction of EAE, as opposed to IgG1 that is associated with Th2 reactivity [23, 24]. These observations imply that the genetic polymorphisms in the locus identified on chromosome 3 may influence the development of Th1-associated autoantibody responses in general, rather than mediating a neurofascin-specific effect, and thereof regulate EAE severity.

## Conclusions

In summary, we demonstrate that epitope spreading to involve neurofascin occurs preferentially in MOG-induced EAE and report that this neurofascin-specific response is associated with increased disease severity and is under genetic regulation. While a role for T cell immunity is well established, the functional significance of antibodies remains controversial. Specifically, it remains to be clarified whether autoantibody responses to specific targets play a significant role in driving disease development directly or reflect ongoing activation of the B cell compartment to provide a pool of antigen presenting cells that exacerbates T cell-mediated inflammation within the CNS. Elucidation of pathophysiological significance of neurofascin-specific antibodies in MOG-induced EAE may clarify this issue and, in addition, permit the stratification of patients into additional subtypes of disease.

## Additional files

Additional file 1: Table S1. EAE clinical characteristics and antibody specific IgG at different time points in MOG-immunized DA rats. (DOCX 28 kb) Additional file 2: Figure S1. Correlations between anti-rrNF IgG and anti-MBP_63–88_ IgG at all time points during MOG-EAE. 31 DA rats were immunized with MOG and anti-rrNF IgG and anti-MBP_63–88_ IgG were assessed in the sera at different time points (day 12, day 26, day 41 and day 56) after immunization by ELISA. Sera were diluted 1:200. The curve was fitted with linear regression using GraphPad Prism 5.0. (PDF 600 kb) Additional file 3: Table S2. EAE clinical characteristics and antibody specific IgG at different time points in MBP_63–88_-immunized DA rats. (DOCX 24 kb) Additional file 4: Figure S2. Correlations between serum samples’ reactivity against different antigens. Rat sera were collected at different time points after MOG immunization. Rat sera were screened for their reactivity against different antigens by ELISA. Correlations between anti-rrNF IgG and a) anti-hNF155 IgG, b) anti-hNF186 IgG, c) anti-CRP IgG and d) anti-IL-9R IgG. Both CRP and IL-9R have been produced in the same murine myeloma NS0 cell line as rrNF. *R* value represents Spearman’s rank correlation coefficient. The curve was fitted with linear regression using GraphPad Prism 5.0. The data were plotted on the same scale in all four graphs to enable comparisons between the graphs. ***p* < 0.01, *****p* < 0.0001. (PDF 695 kb) Additional file 5: Figure S3. Anti-rrNF IgG and anti-TAG-1 IgG before and after anti-rrNF IgG depletion. We depleted anti-rrNF IgG antibodies in 24 serum samples by consecutive adsorptions to plate-bound rrNF and then compared anti-TAG-1 IgG response between intact serum samples and anti-rrNF IgG-depleted serum samples. Data are presented in OD values. The statistical significance of anti-TAG1 IgG between non-depleted and depleted serum samples was calculated using non-parametric Mann-Whitney *U* test in GraphPad Prism 5.0. *****p* < 0.0001. Error bars represent SEM. (PDF 613 kb) Additional file 6: Figure S4. Maximum score (MAX) of 333 DA backcross rats with present or absent anti-rrNF IgG2b antibodies. Anti-rrNF IgG2b (−/+); absence/presence of anti-rrNF IgG2b. MAX; maximum score. The statistical significance between the groups was calculated by using non-parametric Mann-Whitney test in GraphPad Prism 5.0. Error bars represent SEM. (PDF 593 kb)

## Abbreviations

AIL: advanced intercross line
CFA: complete Freund’s adjuvant
MTB: mycobacterium tuberculosis
CNS: central nervous system
CRP: C-reactive protein
CSF: cerebrospinal fluid
DUR: duration of the disease in days
EAE: experimental autoimmune encephalomyelitis
hNF155: human neurofascin 155
hNF186: human neurofascin 186
IFA: incomplete Freund’s adjuvant
IL-9R: interleukin 9 receptor
LOD: logarithm of the odds
MAX: maximum score
MBP: myelin basic protein
MOG: myelin oligodendrocyte glycoprotein
MS: multiple sclerosis
NO: nitric oxide
OCB: oligoclonal bands
OD: optical density
ONS: day of onset of the disease
p.i.: post immunization
PBS: phosphate-buffered saline
QTL: quantitative trait locus
rr: rat recombinant
rrNF: rat recombinant neurofascin
SD: standard deviation
SEM: standard error of the mean
SUM: sum of all scores
TAG-1: contactin-2
WL0: weight change compared to weight at day 0
