# Supplementary material for: MOG-induced experimental autoimmune encephalomyelitis in the rat species triggers anti-neurofascin antibody response that is genetically regulated
Source: J Neuroinflammation. 2015 Oct 29;12:194. doi: 10.1186/s12974-015-0417-2 (PMC4625640; doi:10.1186/s12974-015-0417-2)
Supplement: Additional file 1: Table S1. — EAE clinical characteristics and antibody specific IgG at different time points in MOG-immunized DA rats. (DOCX 28 kb) [file 12974_2015_417_MOESM1_ESM.docx]

**Additional file 1: Table S1. EAE clinical characteristics and antibody specific IgG at different time-points in MOG-immunized DA**

**rats.**

| Immuni-  zation | EAE | MAX | SUM | ONS | DUR | WL0 | anti-rrNF IgG | | | | anti-MBP_63-88_ IgG | | | | anti-MOG IgG | | | |
| --- | --- | --- | --- | --- | --- | --- | --- | --- | --- | --- | --- | --- | --- | --- | --- | --- | --- | --- |
|  |  |  |  |  |  |  | day 12 | day 26 | day 41 | day56 | day 12 | day 26 | day 41 | day 56 | day 12 | day 26 | day 41 | day 56 |
| IFA* | -* | 0* | 0* | 0* | 0* | -3* | 0.006* | 0.012* | 0* | 0* | 0.006* | 0.006* | 0.017* | 0.018* | 0.001* | 0.001* | 0.003* | 0.004* |
| MOG | - | 0 | 0 | 57 | 0 | -6 | - | - | - | - | 0.04 | - | - | - | 0.49 | 0.74 | 0.66 | 0.61 |
| MOG | - | 0 | 0 | 57 | 0 | -3 | - | 0.26 | 0.06 | - | - | - | - | - | 0.36 | 0.92 | 0.57 | 0.58 |
| MOG | + | 4 | 6 | 11 | 2 | 13 | - |  |  |  | - |  |  |  | 0.43 |  |  |  |
| MOG | + | 4 | 9 | 11 | 3 | 16 | - |  |  |  | - |  |  |  | 0.14 |  |  |  |
| MOG | + | 4 | 14 | 10 | 4 | 18 | - |  |  |  | - |  |  |  | 0.49 |  |  |  |
| MOG | + | 4 | 20 | 9 | 7 | 26 | - |  |  |  | 0.06 |  |  |  | 0.15 |  |  |  |
| MOG | + | 4 | 29 | 10 | 11 | 30 | - |  |  |  | - |  |  |  | 0.25 |  |  |  |
| MOG | + | 4 | 33 | 10 | 12 | 29 | - |  |  |  | - |  |  |  | 0.48 |  |  |  |
| MOG | + | 2 | 38 | 21 | 26 | 8 | - | 0.05 | 0.83 | 0.34 | - | - | - | - | 0.29 | 0.71 | 0.87 | 0.73 |
| MOG | + | 1 | 41 | 15 | 41 | 6 | - | 0.76 | - | - | - | - | - | - | 0.18 | 1.14 | 0.91 | 0.80 |
| MOG | + | 4 | 44 | 10 | 15 | 31 | - |  |  |  | - |  |  |  | 0.08 |  |  |  |
| MOG | + | 2 | 44 | 14 | 39 | 8 | 0.05 | - | 0.01 | - | 0.60 | - | - | - | 0.23 | 0.97 | 1.07 | 0.92 |
| MOG | + | 2 | 44 | 19 | 31 | -6 | - | 0.05 | 0.02 | - | 0.04 | - | - | - | 0.40 | 0.43 | 0.85 | 0.84 |
| MOG | + | 5 | 47 | 12 | 16 | 23 | - | - |  |  | - | - |  |  | 0.18 | 0.78 |  |  |
| MOG | + | 3 | 48 | 37 | 24 | 7 | - | 0.13 | 0.12 | - | - | - | - | - | 0.24 | 0.66 | 0.69 | 0.57 |
| MOG | + | 3 | 56 | 10 | 47 | 6 | - | 0.79 | - | - | - | - | 0.78 | 0.24 | 0.45 | 0.87 | 1.10 | 0.95 |
| MOG | + | 5 | 77 | 10 | 28 | 28 | 0.34 | - |  |  | - | - |  |  | 0.53 | 1.03 |  |  |
| MOG | + | 3 | 99 | 14 | 36 | 15 | - | 0.20 | 0.11 | - | - | - | - | - | 0.22 | 1.04 | 0.99 | 0.87 |
| MOG | + | 4 | 102 | 11 | 46 | 16 | 0.06 | 0.19 | 1.05 | 1.09 | - | - | - | - | 0.49 | 0.75 | 1.07 | 0.92 |
| MOG | + | 3 | 103 | 14 | 42 | 16 | - | 0.06 | - | - | 0.04 | - | - | - | 0.46 | 0.95 | 0.85 | 0.76 |
| MOG | + | 4 | 107 | 16 | 38 | 22 | - | 0.76 | 1.11 | 0.75 | - | - | - | - | 0.17 | 0.88 | 0.91 | 0.80 |
| MOG | + | 3 | 107 | 17 | 40 | 21 | 0.03 | 0.20 | 0.16 | - | - | - | - | - | 0.52 | 0.84 | 0.77 | 0.66 |
| MOG | + | 3 | 117 | 17 | 41 | 14 | - | 0.22 | 0.15 | - | 0.06 | 0.09 | 0.10 | - | 0.16 | 0.75 | 0.91 | 0.76 |
| MOG | + | 3 | 131 | 11 | 46 | 24 | - | - | - | - | - | - | - | - | 0.19 | 0.16 | 0.16 | 0.16 |
| MOG | + | 3 | 132 | 11 | 46 | 27 | - | 0.13 | 0.14 | - | 0.05 | 0.44 | 0.13 | 1.61 | 0.50 | 1.09 | 0.95 | 0.78 |
| MOG | + | 3 | 133 | 11 | 46 | 28 | - | - | - | - | - | - | - | - | 0.20 | 1.21 | 1.15 | 0.98 |
| MOG | + | 3 | 136 | 11 | 46 | 23 | - | 0.07 | 0.08 | - | - | 1.50 | 1.02 | 0.28 | 0.20 | 0.87 | 0.97 | 0.82 |
| MOG | + | 3 | 137 | 10 | 47 | 28 | - | - | - | - | - | - | - | - | 0.21 | 0.79 | 0.85 | 0.77 |
| MOG | + | 3 | 138 | 10 | 47 | 23 | - | - | - | - | - | 1.11 | - | - | 0.30 | 1.22 | 0.93 | 0.85 |
| MOG | + | 4 | 140 | 8 | 49 | 18 | - | 0.92 | 0.24 | 0.04 | - | - | - | - | 0.18 | 0.92 | 0.77 | 0.74 |
| MOG | + | 3 | 143 | 7 | 49 | 27 | - | 0.07 | 0.86 | 0.65 | - | - | - | - | 0.24 | 0.95 | 0.82 | 0.74 |

*Immunization of DA rats either with IFA (used as control) or MOG in IFA.*

*EAE -/+; healthy/sick*

*MAX; max score*

*SUM; sum of all scores*

*ONS; day of onset of the disease*

*DUR; duration of the disease in days*

*WL0; weight change compared to weight at day 0 (in %)*

*-; absence of specific IgG*

**; mean value of 9 DA rats immunized with IFA*

*Blank fields; not tested due to death of the animal*

*Antibody specific IgG levels, shown here in OD values, were considered positive when the OD exceeded the cut-off value, which was set at 5 SD above the mean OD in serum specimens from IFA immunized DA rats. The calculated cut-off values are: anti-rrNF IgG; 0.029 for day 12 p.i, 0.039 for day 26 p.i and 0 for the days 41 p.i and 56 p.i., anti-MBP_63-88_ IgG; 0.038 for day 12 p.i, 0.056 for day 26 p.i, 0.07 for day 41 p.i and 0.117 for day 56 p.i. and anti-MOG IgG; 0.011 for day 12 p.i, 0.014 for day 26 p.i, 0.03 for day 41 p.i and 0.032 for day 56 p.i.*

*Individual animals were listed in the table in order of increasing SUM.*
