# Supplementary figures and images for: MOG-induced experimental autoimmune encephalomyelitis in the rat species triggers anti-neurofascin antibody response that is genetically regulated
Source: J Neuroinflammation. 2015 Oct 29;12:194. doi: 10.1186/s12974-015-0417-2 (PMC4625640; doi:10.1186/s12974-015-0417-2)

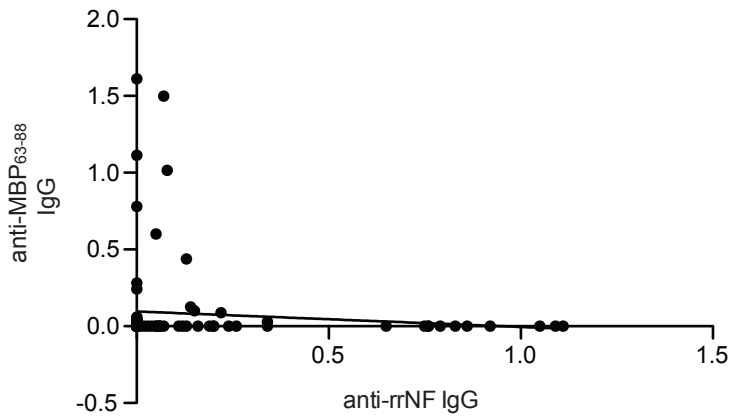

Supplement: Additional file 2: Figure S1. — Correlations between anti-rrNF IgG and anti-MBP63–88 IgG at all time points during MOG-EAE. 31 DA rats were immunized with MOG and anti-rrNF IgG and anti-MBP63–88 IgG were assessed in the sera at different time points (day 12, day 26, day 41 and day 56) after immunization by ELISA. Sera were diluted 1:200. The curve was fitted with linear regression using GraphPad Prism 5.0. (PDF 600 kb) [file 12974_2015_417_MOESM2_ESM.pdf]

**a**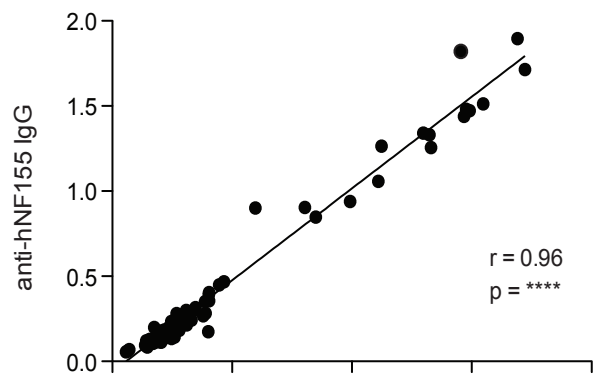**b**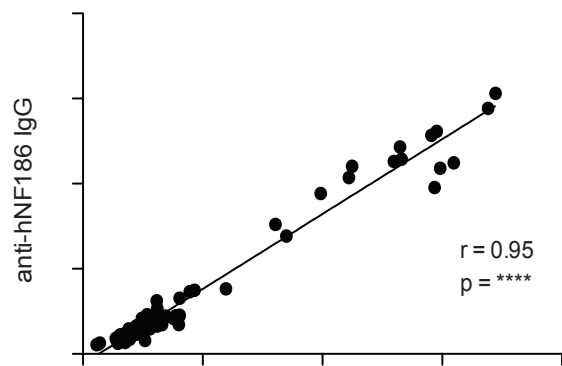**c**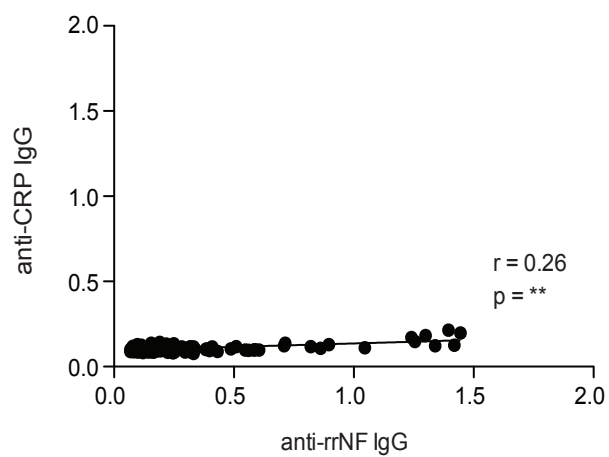**d**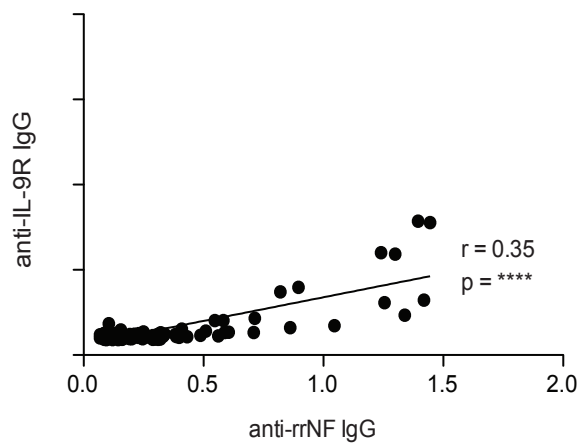

Supplement: Additional file 4: Figure S2. — Correlations between serum samples’ reactivity against different antigens. Rat sera were collected at different time points after MOG immunization. Rat sera were screened for their reactivity against different antigens by ELISA. Correlations between anti-rrNF IgG and a) anti-hNF155 IgG, b) anti-hNF186 IgG, c) anti-CRP IgG and d) anti-IL-9R IgG. Both CRP and IL-9R have been produced in the same murine myeloma NS0 cell line as rrNF. R value represents Spearman’s rank correlation coefficient. The curve was fitted with linear regression using GraphPad Prism 5.0. The data were plotted on the same scale in all four graphs to enable comparisons between the graphs. **p < 0.01, ****p < 0.0001. (PDF 695 kb) [file 12974_2015_417_MOESM4_ESM.pdf]

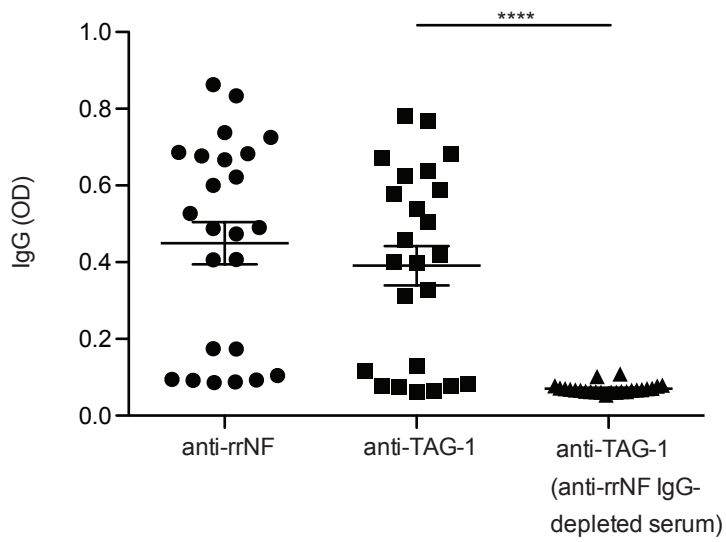

Supplement: Additional file 5: Figure S3. — Anti-rrNF IgG and anti-TAG-1 IgG before and after anti-rrNF IgG depletion. We depleted anti-rrNF IgG antibodies in 24 serum samples by consecutive adsorptions to plate-bound rrNF and then compared anti-TAG-1 IgG response between intact serum samples and anti-rrNF IgG-depleted serum samples. Data are presented in OD values. The statistical significance of anti-TAG1 IgG between non-depleted and depleted serum samples was calculated using non-parametric Mann-Whitney U test in GraphPad Prism 5.0. ****p < 0.0001. Error bars represent SEM. (PDF 613 kb) [file 12974_2015_417_MOESM5_ESM.pdf]

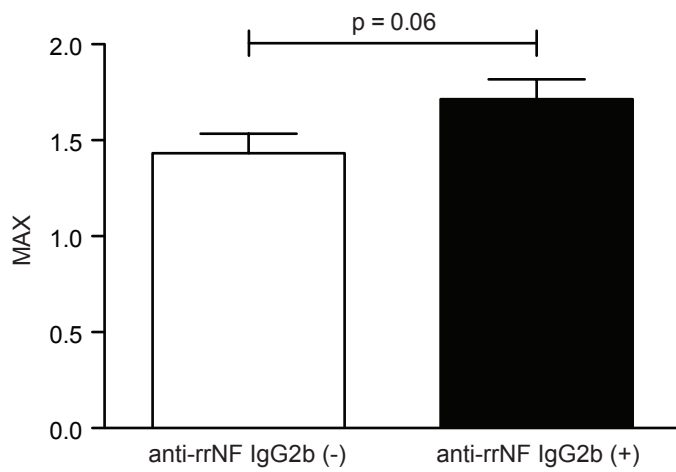

Supplement: Additional file 6: Figure S4. — Maximum score (MAX) of 333 DA backcross rats with present or absent anti-rrNF IgG2b antibodies. Anti-rrNF IgG2b (−/+); absence/presence of anti-rrNF IgG2b. MAX; maximum score. The statistical significance between the groups was calculated by using non-parametric Mann-Whitney test in GraphPad Prism 5.0. Error bars represent SEM. (PDF 593 kb) [file 12974_2015_417_MOESM6_ESM.pdf]
