# Supplementary material for: MOG-induced experimental autoimmune encephalomyelitis in the rat species triggers anti-neurofascin antibody response that is genetically regulated
Source: J Neuroinflammation. 2015 Oct 29;12:194. doi: 10.1186/s12974-015-0417-2 (PMC4625640; doi:10.1186/s12974-015-0417-2)
Supplement: Additional file 3: Table S2. — EAE clinical characteristics and antibody specific IgG at different time points in MBP63–88-immunized DA rats. (DOCX 24 kb) [file 12974_2015_417_MOESM3_ESM.docx]

**Additional file 3: Table S2. EAE clinical characteristics and antibody specific IgG at different time-points in MBP_63-88_-immunized DA**

**rats.**

| Immuni-  zation | EAE | MAX | SUM | ONS | DUR | WL0 | anti-rrNF IgG | | | | anti-MBP_63-88_ IgG | | | | anti-MOG IgG | | | |
| --- | --- | --- | --- | --- | --- | --- | --- | --- | --- | --- | --- | --- | --- | --- | --- | --- | --- | --- |
|  |  |  |  |  |  |  | day 12 | day 26 | day 41 | day56 | day 12 | day 26 | day 41 | day 56 | day 12 | day 26 | day 41 | day 56 |
| CFA* | -* | 0* | 0* | 0* | 0* | -5* | 0* | 0* | 0.01* | 0* | 0.0008* | 0.0008* | 0.0002* | 0.003  * | 0.048* | 0.032* | 0.028* | 0.028* |
| MBP | + | 2 | 13 | 11 | 9 | 12 | - | - | - | - | 0.11 | 0.76 | 0.70 | 0.76 | - | - | - | - |
| MBP | + | 2 | 13 | 11 | 9 | 13 | - | - | - | - | 0.19 | 0.83 | 0.75 | 0.54 | - | - | - | - |
| MBP | + | 3 | 14 | 11 | 9 | 12 | - | - | - | - | 0.19 | 0.56 | 0.46 | 0.37 | 0.29 | 1.44 | 1.24 | 1.01 |
| MBP | + | 3 | 15 | 12 | 10 | 16 | - | - | - | - | 0.16 | 0.47 | 0.54 | 0.42 | - | - | - | - |
| MBP | + | 3 | 16 | 11 | 11 | 16 | - | - | - | - | 0.04 | 0.32 | 0.24 | 0.18 | - | - | - | - |
| MBP | + | 3 | 16 | 13 | 7 | 16 | 0.09 | - | - | - | 0.12 | 0.65 | 0.56 | 0.36 | - | - | - | - |
| MBP | + | 2 | 17 | 12 | 11 | 11 | - | - | - | - | 0.34 | 0.57 | 0.50 | 0.51 | - | - | - | - |
| MBP | + | 2 | 18 | 12 | 11 | 13 | - | - | - | - | 0.13 | 0.95 | 0.95 | 0.76 | - | - | - | - |
| MBP | + | 3 | 19 | 10 | 10 | 13 | - | - | - | - | 0.08 | 0.66 | 0.76 | 0.66 | - | 1.20 | 0.99 | 0.75 |
| MBP | + | 3 | 21 | 11 | 16 | 18 | - | - | - | - | 0.37 | 0.84 | 0.98 | 0.90 | - | - | - | - |
| MBP | + | 2 | 21 | 11 | 14 | 13 | - | - | - | - | 0.27 | 0.76 | 0.75 | 0.71 | - | - | - | - |
| MBP | + | 3 | 25 | 11 | 16 | 17 | - | - | - | - | 0.29 | 1.08 | 1.00 | 0.76 | - | - | 0.13 | 0.17 |
| MBP | + | 3 | 26 | 8 | 19 | 23 | - | - | - | - | 0.27 | 0.85 | 1.08 | 0.89 | - | - | - | - |
| MBP | + | 3 | 26 | 10 | 21 | 23 | - | - | - | - | 0.17 | 0.45 | 0.47 | 0.38 | - | - | - | - |
| MBP | + | 3 | 27 | 9 | 18 | 18 | - | - | - | - | 0.58 | 1.07 | 0.98 | 0.81 | - | - | - | - |
| MBP | + | 3 | 28 | 8 | 21 | 20 | - | - | - | - | 0.59 | 1.02 | 1.09 | 0.96 | - | - | 0.10 | 0.10 |
| MBP | + | 3 | 28 | 8 | 22 | 21 | - | - | - | - | 0.40 | 0.96 | 0.86 | 0.73 | - | - | - | - |
| MBP | + | 3 | 29 | 8 | 22 | 19 | - | - | - | - | 0.41 | 0.83 | 0.87 | 0.77 | - | - | - | - |
| MBP | + | 3 | 30 | 9 | 21 | 21 | - | - | - | - | 0.33 | 1.13 | 1.20 | 1.17 | - | - | - | - |
| MBP | + | 3 | 30 | 8 | 25 | 24 | - | - | - | - | 0.19 | 0.94 | 0.94 | 0.88 | - | - | - | - |
| MBP | + | 3 | 30 | 8 | 24 | 24 | - | - | - | - | 0.11 | 0.86 | 0.69 | 0.57 | - | - | - | - |
| MBP | + | 3 | 32 | 9 | 26 | 22 | - | - | - | - | 0.25 | 0.52 | 0.33 | 0.22 | - | - | - | - |
| MBP | + | 3 | 36 | 8 | 25 | 21 | - | - | - | - | 0.39 | 1.07 | 1.08 | 0.94 | - | - | - | - |
| MBP | + | 3 | 37 | 10 | 27 | 23 | - | - | - | - | 0.36 | 0.61 | 0.48 | 0.30 | - | - | - | - |
| MBP | + | 3 | 38 | 8 | 23 | 23 | - | - | - | - | 0.46 | 0.75 | 0.72 | 0.58 | - | - | - | - |

*Immunization of DA rats either with CFA (used as control) or MBP_63-88_* *in CFA.*

*EAE -/+; healthy/sick*

*MAX; max score*

*SUM; sum of all scores*

*ONS; day of onset of the disease*

*DUR; duration of the disease in days*

*WL0; weight change compared to weight at day 0 (in %)*

*-; absence of specific IgG*

**; mean value of 9 DA rats immunized with CFA*

*Blank fields; not tested due to death of the animal*

*Antibody specific IgG levels, shown here in OD values, were considered positive when the OD exceeded the cut-off value, which was set at 5 SD above the mean OD in serum specimens from CFA immunized DA rats. The calculated cut-off values are: anti-rrNF IgG; 0 for the days 12 p.i, 26 p.i and 56 p.i and 0.018 for day 41 p.i., anti- MBP_63-88_ IgG; 0.009 for day 12 p.i, 0.008 for day 26 p.i, 0.004 for day 41 p.i and 0.005 for day 56 p.i. and anti-MOG IgG; 0.277 for day 12 p.i, 0.135 for day 26 p.i, 0.087 for day 41 p.i and 0.071 for day 56 p.i.*
